# Levels of Murine, but Not Human, CXCL13 Are Greatly Elevated in NOD-SCID Mice Bearing the AIDS-Associated Burkitt Lymphoma Cell Line, 2F7

**DOI:** 10.1371/journal.pone.0072414

**Published:** 2013-08-02

**Authors:** Daniel P. Widney, Tove Olafsen, Anna M. Wu, Christina M. R. Kitchen, Jonathan W. Said, Jeffrey B. Smith, Guadalupe Peña, Larry I. Magpantay, Manuel L. Penichet, Otoniel Martinez-Maza

**Affiliations:** 1 Department of Obstetrics & Gynecology, David Geffen School of Medicine at UCLA, University of California Los Angeles, Los Angeles, California, United States of America; 2 Department of Microbiology, Immunology and Molecular Genetics, David Geffen School of Medicine at UCLA, University of California, Los Angeles, California, United States of America; 3 Department of Molecular and Medical Pharmacology, David Geffen School of Medicine at UCLA, University of California Los Angeles, Los Angeles, California, United States of America; 4 Department of Pathology and Laboratory Medicine, David Geffen School of Medicine at UCLA, University of California Los Angeles, Los Angeles, California, United States of America; 5 Department of Pediatrics, David Geffen School of Medicine at UCLA, University of California Los Angeles, Los Angeles, California, United States of America; 6 Department of Surgery, David Geffen School of Medicine at UCLA, University of California Los Angeles, Los Angeles, California, United States of America; 7 Department of Biostatistics, UCLA Fielding School of Public Health, University of California Los Angeles, Los Angeles, California, United States of America; 8 Department of Epidemiology, UCLA Fielding School of Public Health, University of California Los Angeles, Los Angeles, California, United States of America; 9 UCLA AIDS Institute, University of California Los Angeles, Los Angeles, California, United States of America; 10 Jonsson Comprehensive Cancer Center, University of California Los Angeles, Los Angeles, California, United States of America; 11 California NanoSystems Institute, University of California Los Angeles, Los Angeles, California, United States of America; University of Massachusetts Medical School, United States of America

## Abstract

Currently, few rodent models of AIDS-associated non-Hodgkin’s lymphoma (AIDS-NHL) exist. In these studies, a novel mouse/human xenograft model of AIDS-associated Burkitt lymphoma (AIDS-BL) was created by injecting cells of the human AIDS-BL cell line, 2F7, intraperitoneally into NOD-SCID mice. Mice developed tumors in the peritoneal cavity, with metastases to the spleen, thymus, and mesenteric lymph nodes. Expression of the chemokine receptor, CXCR5, was greatly elevated *in vivo* on BL tumor cells in this model, as shown by flow cytometry. CXCL13 is the ligand for CXCR5, and serum and ascites levels of murine, but not human, CXCL13 showed a striking elevation in tumor-bearing mice, with levels as high as 200,000 pg/ml in ascites, as measured by ELISA. As shown by immunohistochemistry, murine CXCL13 was associated with macrophage-like tumor-infiltrating cells that appeared to be histiocytes. Blocking CXCR5 on 2F7 cells with neutralizing antibodies prior to injection into the mice substantially delayed tumor formation. The marked elevations in tumor cell CXCR5 expression and in murine CXCL13 levels seen in the model may potentially identify an important link between tumor-interacting histiocytes and tumor cells in AIDS-BL. These results also identify CXCL13 as a potential biomarker for this disease, which is consistent with previous studies showing that serum levels of CXCL13 were elevated in human subjects who developed AIDS-lymphoma. This mouse model may be useful for future studies on the interactions of the innate immune system and AIDS-BL tumor cells, as well as for the assessment of potential tumor biomarkers for this disease.

## Introduction

The most common subtypes of AIDS-associated non-Hodgkin’s lymphoma (AIDS-NHL) are Burkitt lymphoma (BL), diffuse large B cell lymphoma (DLBCL), and primary central nervous system lymphoma (PCNSL) [1,2]. It is thought that many of these tumors result from hyperactivation of B cells, which occurs in HIV infection and can contribute to genetic damage that leads to tumorigenesis [3]. Work by McGrath et al. suggests that tumor-infiltrating cells play an important role in AIDS-lymphoma pathogenesis [4–6]. Specifically, about half of AIDS-NHLs were seen to contain tumor-associated macrophages (TAM), many of which appeared to be infected with HIV strains that were resistant to combination anti-retroviral therapy (cART) [4,7]. Furthermore, macrophages from human AIDS-lymphomas of the more rare primary effusion lymphoma (PEL) subtype were shown to be able to induce lymphoma formation when injected into immunodeficient SCID mice [6]. In this case, the induced tumors appeared to be T cell lymphomas of murine origin; however, the lymphomagenic potential of these macrophages was clear.

CXCL13 (BLC, BCA-1) is a chemokine most known for regulating the homeostatic movement of mature B cells through secondary lymphoid tissue [8]. It can also be induced during certain types of inflammatory processes, such as rheumatoid arthritis and Sjögren’s syndrome, where it aids in the formation of ectopic lymphoid tissues, and thus promotes the disease process [9,10]. Recently, we demonstrated that serum levels of CXCL13 are substantially increased during HIV infection [11]. The receptor for CXCL13 is CXCR5 (BLR1) [8], and it has been shown that levels of CXCR5 are significantly decreased on the surface of circulating B cells during HIV infection, and that these cells, in contrast to B cells from healthy individuals, express CXCL13 [12,13]. These results suggest that CXCL13 could potentially play a role in the B cell hyperactivation observed during HIV infection that is believed to contribute to AIDS-NHL formation.

CXCL13 has been more directly implicated in the biology of some B cell tumors, including several non-HIV-associated lymphomas, such as follicular lymphoma and primary intraocular lymphoma [14,15]. In the case of primary intraocular lymphoma, tumor cells expressed CXCR5, and adjacent non-cancerous ocular cells expressed CXCL13, suggesting that these ocular cells might be directing tumor growth [14]. In other lymphomas, CXCL13 induced chemotaxis of tumor cells [16,17]. Recently, we showed that serum levels of CXCL13 were elevated in preceding AIDS-NHL diagnosis [18]. Furthermore, CXCR5 and/or CXCL13 were expressed in most primary AIDS-NHL tumor specimens. Several AIDS-NHL cell lines, including the AIDS-BL cell line, 2F7, also demonstrated chemotaxis towards CXCL13 *in vitro* [18].

As few mouse models of AIDS-lymphoma currently exist, our aim in these studies was to create a mouse/human xenograft model of AIDS-BL and to evaluate CXCR5 and CXCL13 expression in this model. Tumors readily formed intra-abdominally in NOD-SCID mice after intraperitoneal (i.p.) injection of cells of the AIDS-BL cell line, 2F7. Furthermore, cells of AIDS-BL tumors growing in the mice showed greatly elevated surface expression of CXCR5. High levels of murine, but not human, CXCL13, also were seen in these animals, and tumors contained tumor-infiltrating cells that stained positively for murine CXCL13 by immunohistochemistry.

## Materials and Methods

### Ethics statement

The AIDS-lymphoma cell lines, 2F7, R, and BCBL-1 are of human origin, but are long-established cell lines that have previously been described in the literature and that were obtained commercially or from other sources without any information that would identify the subjects from whom they were derived [20]. This study, therefore, did not involve the use of human subjects, and was not subject to Institutional Review Board (IRB) review. All animal experiments were carried out in strict accordance with the Policy on Humane Care and Use of Laboratory Animals of the United States Public Health Service. The protocol was approved by the Animal Research Committee (ARC) at UCLA (Assurance # A3196-01). Isoflurane was used for euthanasia and to anesthetize animals during i.p. injections of tumor cells and when restraint of animals was needed for PET imaging. All efforts were made to minimize suffering.

### Cell lines

The AIDS-BL cell line, 2F7, was obtained from the American Type Culture Collection (ATCC; Rockville, MD), and has been previously described [19]. It was derived from an AIDS-BL, and is positive for the Epstein–Barr virus (EBV), negative for HIV, and expresses the B cell markers, CD19 and CD20, but not the CD3 T cell marker, on its cell surface [19,20]. This cell line was cultured in standard media as previously described [20]. The AIDS-lymphoma cell lines, R (AIDS-DLBCL subtype), and BCBL-1 (primary effusion lymphoma [AIDS-PEL] subtype), were obtained and cultured in standard media as described [20]. The R cell line is EBV(+), whereas the BCBL-1 cell line is EBV(-), but positive for human herpesvirus-8 (HHV-8) [20].

### Flow cytometry

Cell surface expression of the chemokine receptor, CXCR5, was examined by flow cytometry using an indirect staining protocol as previously described [18,20]. Briefly, cells were incubated with a rat IgG_2b_ anti-CXCR5 antibody; as a control, other cells were incubated with a rat IgG_2b_ isotype control antibody. Samples were then incubated with a phycoerythrin (PE)-conjugated goat anti-rat IgG secondary antibody, and cell fluorescence was measured by flow cytometry. During data collection, at least 5,000 cells/events were acquired per tube/condition. Dead cells were excluded from the analysis using forward- and side-scatter. For all flow cytometry studies, data were acquired using a Becton Dickinson LSR machine, and analyzed using CellQuest Pro 5.1 software (Becton Dickinson, Franklin Lakes, New Jersey, USA).

### Animals

Immunodeficient female NOD-SCID (NOD/LzSz-Prkdc(SCID)/J) mice, 8-12 weeks of age, were purchased from The Jackson Laboratory (Bar Harbor, Maine, USA).

### In vivo studies

To create a mouse/human xenograft model of AIDS-BL, 10^6^ 2F7 cells in saline were injected i.p. into mice. Control animals were injected with saline alone. Mice were also injected i.p. with 10^6^ cells of the R cell line (AIDS-DLBCL), or of the BCBL-1 cell line (AIDS-PEL). Typically, mice developed palpable tumors in the abdomen at 5-10 weeks after inoculation with the cell lines. These tumors were reducible to single-cell suspensions when placed in culture media. After filtering through 100-µm pore size cell strainers (Becton Dickinson), and washing in media to remove debris, cells could readily be used in *in vitro* studies. For CXCR5 neutralization experiments in the 2F7 AIDS-BL model, a monoclonal anti-human CXCR5 antibody (Clone 51505, mouse IgG_2B_) with known neutralizing activity to CXCR5 was obtained in a low-endotoxin format from R&D Systems (Minneapolis, Minnesota, USA). An isotype control antibody was also obtained from R&D Systems (Clone 20116, mouse IgG_2B_). For these experiments, cultured 2F7 cells (10^6^ cells/ml) were incubated at 37^°^ C in the presence of 10 µg/ml of neutralizing or control antibody for 40 minutes. Cells were then centrifuged and resuspended in sterile pharmaceutical-grade saline, and 10^6^ cells were injected i.p. into NOD-SCID mice as per the standard model. Mice were then monitored for tumor development.

### Imaging of animals

Imaging studies of animals injected with 2F7 cells were performed in the Crump Institute for Molecular Imaging at UCLA. For positron emission tomography (PET) imaging, mice were first injected via the tail vein with 200-300 µCi of [^18^F]-Fluorodeoxyglucose (FDG). After 60 minutes, mice were then scanned over a 10-minute period using a Focus 220 microPET scanner (Siemens Preclinical Solutions, Knoxville, Tennesse, USA). Finally, MicroCT scanning was performed for 10 minutes using a microCAT II (Concorde Microsystems, Knoxville, Tennessee, USA) instrument. The AMIDE software program (Version 0.9.2) was used to co-register PET and CT scans to create a single image for each mouse, as previously described [21,22].

### Determination of CXCL13 levels

Levels of murine and human CXCL13 in serum and in tumor ascites fluid were determined by ELISA, using kits validated to be specific for either murine or human CXCL13 (Quantikine™, R&D Systems), with no cross-reactivity. The manufacturer’s protocols were followed in performing the assays. The lower limit of detection of the murine CXCL13 assay was 15.6 pg/ml; for human CXCL13 the limit was 7.8 pg/ml.

### Estimation of murine CXCL13 levels in the peritoneal fluid of healthy NOD-SCID mice

The abdominal cavities of healthy control female NOD-SCID mice (n = 7) were lavaged using serum-free RPMI-1640 medium. Lavage fluid was then concentrated using a protocol similar to one that has been previously described [23]. Briefly, lavage fluid from animals was pooled to give a total volume of 20 ml, and then concentrated 25-fold using an Amicon Ultra-15 centrifugal unit (Millipore, Billerica, Massachusetts, USA) designed to retain molecules with a mass greater than 3 KDa. The concentrate was then stored at -80^°^ C until further use.

To estimate background murine CXCL13 levels in the peritoneal fluid of these mice, levels of murine CXCL13 were then measured in this concentrate by ELISA, and determined to be 3942 pg/ml. The estimated level of murine CXCL13 in the peritoneal cavity prior to lavage was calculated using the formula: ((3942 pg/ml)/25) x (20 ml total pooled lavage fluid/7 mice)) / (estimated volume of peritoneal fluid in each mouse prior to lavage). As the actual volume of peritoneal fluid in these mice was very small, it was not possible to measure it directly prior to lavage. Assuming a peritoneal fluid volume of 0.05 ml per mouse led to an estimated murine CXCL13 level of ~9,000 pg/ml in peritoneal fluid, while assuming an average volume of 0.5 ml led to an estimated CXCL13 concentration of ~900 pg/ml.

### Immunohistochemistry

Immunohistochemistry studies on tumors from mice injected with 2F7 cells were performed on formalin-fixed, paraffin-embedded tumor sections by the Translational Pathology Core Laboratory in the Department of Pathology & Laboratory Medicine at UCLA. For staining for human CXCR5, murine CXCL13, and human CXCL13, sections (4 µm thickness) were first processed as previously described [18], except that the wash buffer used was phospho-buffered saline with Tween (PBST). These processing steps included antigen retrieval treatments and treatments to quench endogenous peroxidase. For CXCR5, sections were stained using identical reagents as previously described [18], except that the primary antibody staining step was followed by a 30 minute incubation with a secondary biotinylated polyclonal rabbit anti-rat immunoglobulin antibody reagent (Cat. #E0468, Dako, Carpinteria, California, USA) at a 1:200 dilution. The signal was detected using the rabbit horseradish peroxidase EnVision kit (DAKO), which included the use of anti-rabbit HRP polymer and diaminobenzidine (Cat. # 32741, Sigma-Aldrich, St. Louis, Missouri, USA) as the reaction substrate. The sections were counterstained with hematoxylin. As a control, serial sections were incubated with an isotype control antibody as previously noted [18]. Serial sections were also stained with hematoxylin and eosin (H&E).

For human CXCL13, sections were incubated with primary antibody as previously described [18]. A similar protocol was used for murine CXCL13, except that the primary antibody was a specific polyclonal goat IgG (R&D Systems), used at a concentration of 15 µg/ml. Normal goat IgG (Santa Cruz Biotechnology, Santa Cruz, California, USA) was used as a control. Subsequent steps in the staining protocol for both human and murine CXCL13 were identical to those for CXCR5 (above).

For F4/80 (murine histiocyte marker) staining, endogenous peroxidase activity was first blocked with 3% hydrogen peroxide in methanol for 10 minutes. Next, proteolytic induced epitope retrieval (PIER) was carried out with proteinase K (Cat. #S3020, Dako) for 10 minutes at 37^°^ C. Sections were then stained overnight at 4^°^ C with monoclonal rat anti-mouse F4/80 (Cat. # MCA497b, clone CI:A3-1, IgG_2b_, AbD Serotec, Raleigh, North Carolina, USA) at a dilution of 1:50. Subsequent steps were identical to those in the CXCR5 staining protocol (above).

All stained slides were scored and photographed by a pathologist (JWS). Pictures were taken using an Olympus DP-25 camera attached to an Olympus BX51 bright field microscope, and recorded on a Smart Media digital card. The 20X objective lens (UPlan Apo, Japan) used to take the pictures had an aperture of 0.70. Pictures were edited for publication using Canvas 5.0.3 (ACD Systems of America, Inc., Miami, Florida, USA).

### Statistical analysis

The statistical significance of differences in CXCL13 levels between two experimental groups was evaluated using a 2-tailed Student’s *t*-test. If variances between groups were unequal, then the unequal-variances form of the test was used. Data were log-transformed to meet model assumptions, although they are given in linear scale for ease of presentation. Pearson product moment correlation was used to assess the correlation between mCXCL13 levels in the serum and in the ascites of mice with tumors. For survival studies of mice with tumors, survival rates in different experimental groups were compared using the method of Kaplan-Meier and the log-rank test. All analyses were conducted using SAS Version 9.2 (SAS Institute, Inc., Cary, North Carolina, USA).

## Results

### Tumors consistently form in NOD-SCID mice inoculated with the human AIDS-NHL cell lines, 2F7, R and BCBL-1

**Figure 1:**
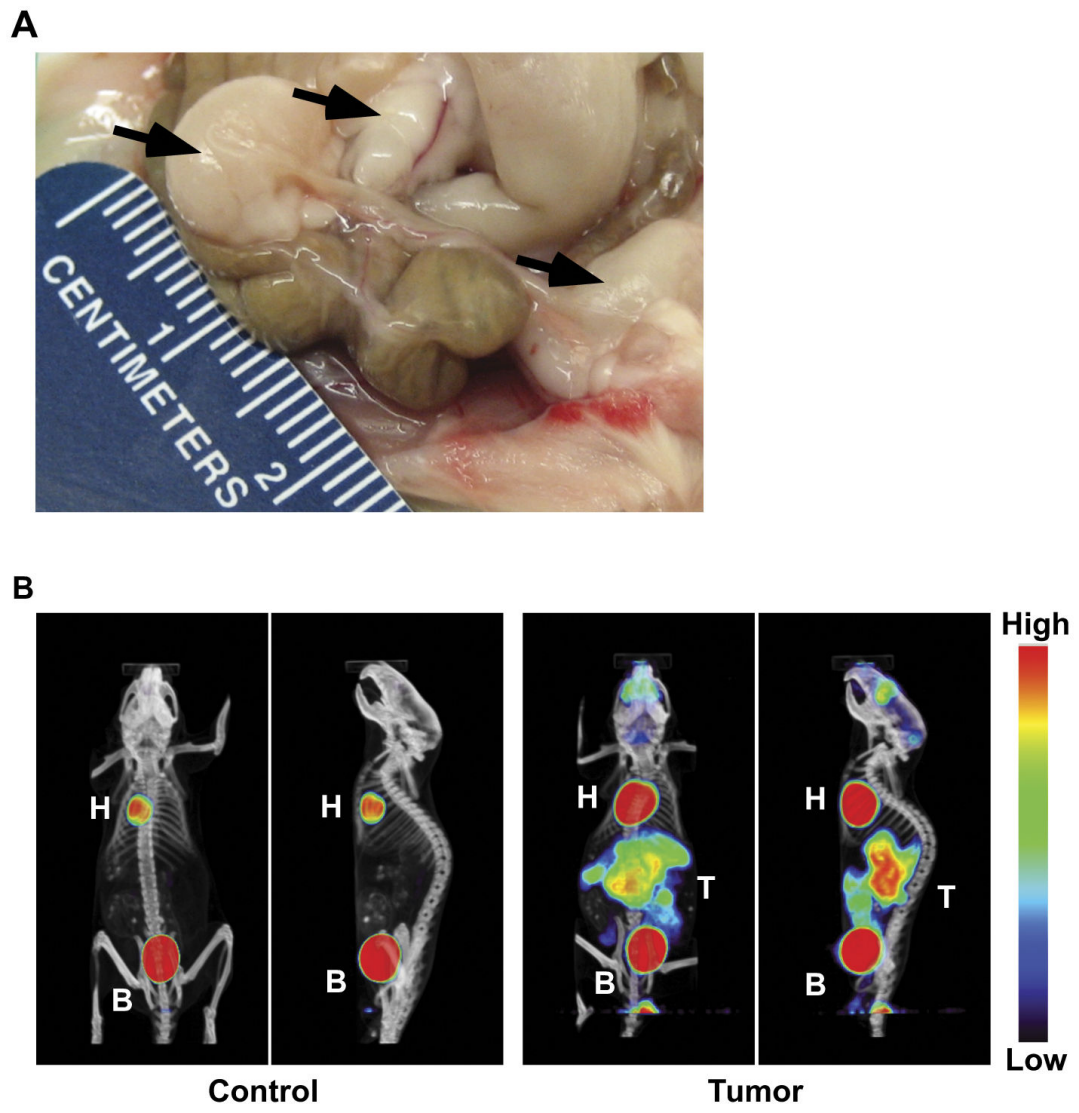
Images of 2F7-derived AIDS-Burkitt lymphoma (BL) tumors in NOD-SCID mice. (**A**) A picture showing three tumors growing in the peritoneal cavity, as indicated by arrows. The left tumor is located on an ovary; the middle tumor is in the mesentery, and the tumor to the right is under the abdominal wall. Brown areas are intestines. (**B**) PET images of a healthy control mouse that was not inoculated with tumor cells (left two panels), and of a mouse with advanced peritoneal tumors (right two panels). Animals were imaged 1 hour after i.v. injection of [^18^F]-FDG. Coronal and sagittal images are shown. The color scale indicates the relative intensity of radioactivity levels, with black representing the lowest level and red the highest. H = heart; T = tumor masses; B = bladder.

**Figure 2:**
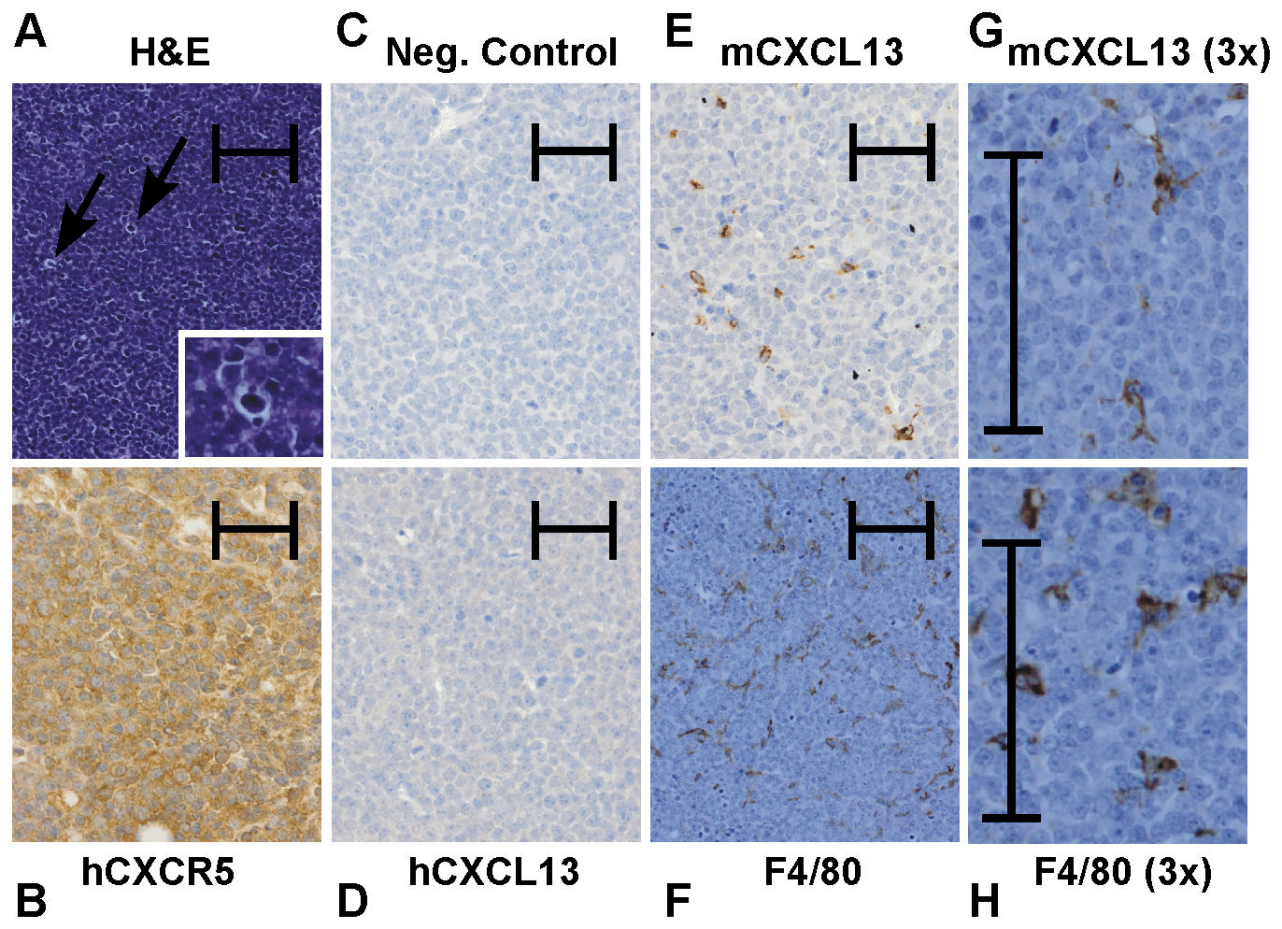
Expression of CXCR5 and CXCL13 on 2F7-derived AIDS-BL tumor cells in NOD-SCID mice, by immunohistochemistry. Immunohistochemistry was performed as described in the *Materials and Methods*, using an HRP/DAB color development system with hematoxylin counterstaining. Some sections were stained with both hematoxylin and eosin (H and E). Typical staining results for each marker are given in the figure as follows: (**A**) H and E, (**B**) human CXCR5, (**C**) a representative negative control, (**D**) human CXCL13, (**E**) murine CXCL13, and (**F**) F4/80. The results of the H&E staining in (**A**) show a tumor that in many respects resembles human BL, which typically contain significant numbers of infiltrating macrophages, giving a “starry sky” appearance (see arrows). The corner insert in (**A**) shows a 3-fold enlargement of the area in the image to which the upper arrow points, with such a macrophage in the center of the enlargement. The image in (**E**) shows that tumors appear to be infiltrated with scattered cells that stain positive for murine CXCL13. In an attempt to more precisely determine the cell type of these infiltrating cells serial tumor sections were stained separately for murine CXCL13 and for F4/80, and images of the resulting staining were enlarged 3-fold compared to the images in (**A**–**F**) to provide more detail (**G**, **H**). In (**G**) and (**H**), both mCXCL13(+) and F4/80(+) cells appear to be large cells that sometimes have dendritic processes. All images shown in this figure were taken at an original magnification of 200X. Each image contains an internal scale marker that is 100 microns in length.

Mice consistently developed tumors within the peritoneal cavity about 8-10 weeks after i.p. injection of 10^6^ 2F7 cells (Figure 1). Metastases to the spleen, thymus, and mesenteric lymph nodes were common in this model (not shown). Tumors bore a significant resemblance to human BL on histopathology examination in that they were made up of intermediate-sized cells that contained round oval nuclei, distinct nucleoli, and had a blast-like appearance (Figure 2A). Scattered “starry sky” macrophages were present (Figure 2A). When examined using flow cytometry, tumor cells from both primary tumors and metastases uniformly expressed the human pan-B cell markers, CD19 and CD20, on their cell surface, similar to cells of the 2F7 cell line grown *in vitro* [19,20] (not shown). Tumor cells were also occasionally present in the brains of some animals, as cells positive for human CD19 and human CD20 were sometimes present in cell suspensions from the brains of animals with intra-abdominal primary 2F7-derived tumors, as demonstrated by flow cytometry (data not shown). Such cells were never present in the brains of control animals (not shown). PET images of mice with tumors also were occasionally suggestive of brain metastases (see Figure 1, for example). However, more detailed studies of possible brain metastases were not performed.

Mice injected i.p with R cells (AIDS-DLCBL subtype) also developed intra-abdominal tumors, whereas several mice injected with BCBL-1 cells (AIDS-PEL subtype) formed intra-abdominal effusions (data not shown). However, extensive studies of R- and BCBL-1-derived tumors were not performed.

### 2F7-derived tumors express greatly elevated levels of the chemokine receptor, CXCR5

**Figure 3:**
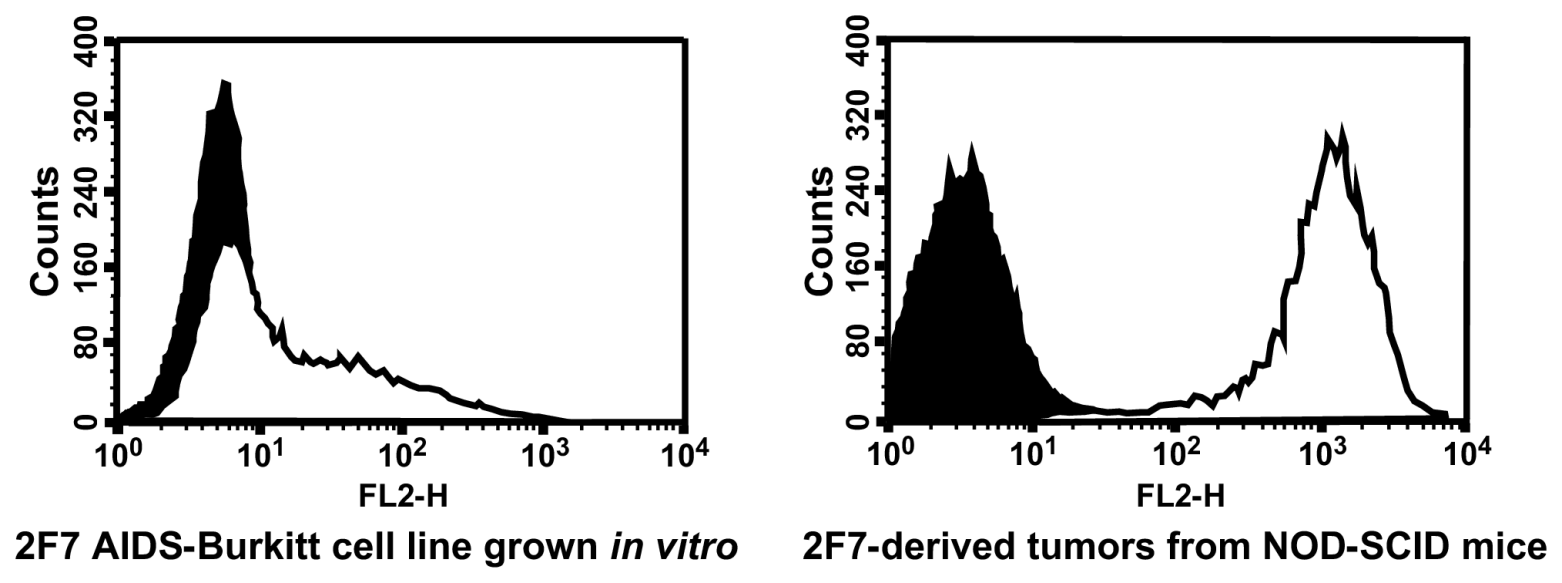
CXCR5 expression is greatly elevated on cells of 2F7-derived AIDS-BL tumors in NOD-SCID mice. The left panel shows CXCR5 expression, as determined by flow cytometry, on cells of the AIDS-BL cell line, 2F7, growing in culture; the right panel shows expression on 2F7-derived tumors growing in NOD-SCID mice. Data are presented as histograms, with relative fluorescence intensity given on the x-axis and cell counts given on the y-axis. For both panels, cells were stained for CXCR5 expression using an indirect staining protocol, as noted in the *Materials and Methods*; the secondary antibody was labeled with phycoerythrin (PE). Open histograms show staining for CXCR5; closed histograms show staining with isotype control antibody.

The 2F7 cell line, when grown in culture, showed modest surface expression of human CXCR5 (Figure 3). In contrast, cells isolated from *in vivo* tumors derived from 2F7 cells showed greatly elevated levels of CXCR5 expression (Figure 3). This striking elevation in CXCR5 expression was seen uniformly on all tumors in these mice, both primary (Figure 3) and metastatic (not shown). Immunohistochemistry studies (Figure 2B-C) also confirmed the expression of human CXCR5 by tumor cells.

### Levels of murine, but not human, CXCL13 are greatly elevated in both the serum and ascites of mice with 2F7-derived AIDS-BL tumors

To assess human CXCL13 levels in the 2F7 AIDS-BL model, serum and tumor ascites were collected from seven mice that had end-stage tumors, and CXCL13 levels were then measured using ELISA specific for human or murine CXCL13. In all cases, no human CXCL13 was detected (not shown). Similar results were seen in serum or ascites fluid from the animals that developed tumors after being inoculated with the R or BCBL-1 cell lines (data not shown).

**Figure 4:**
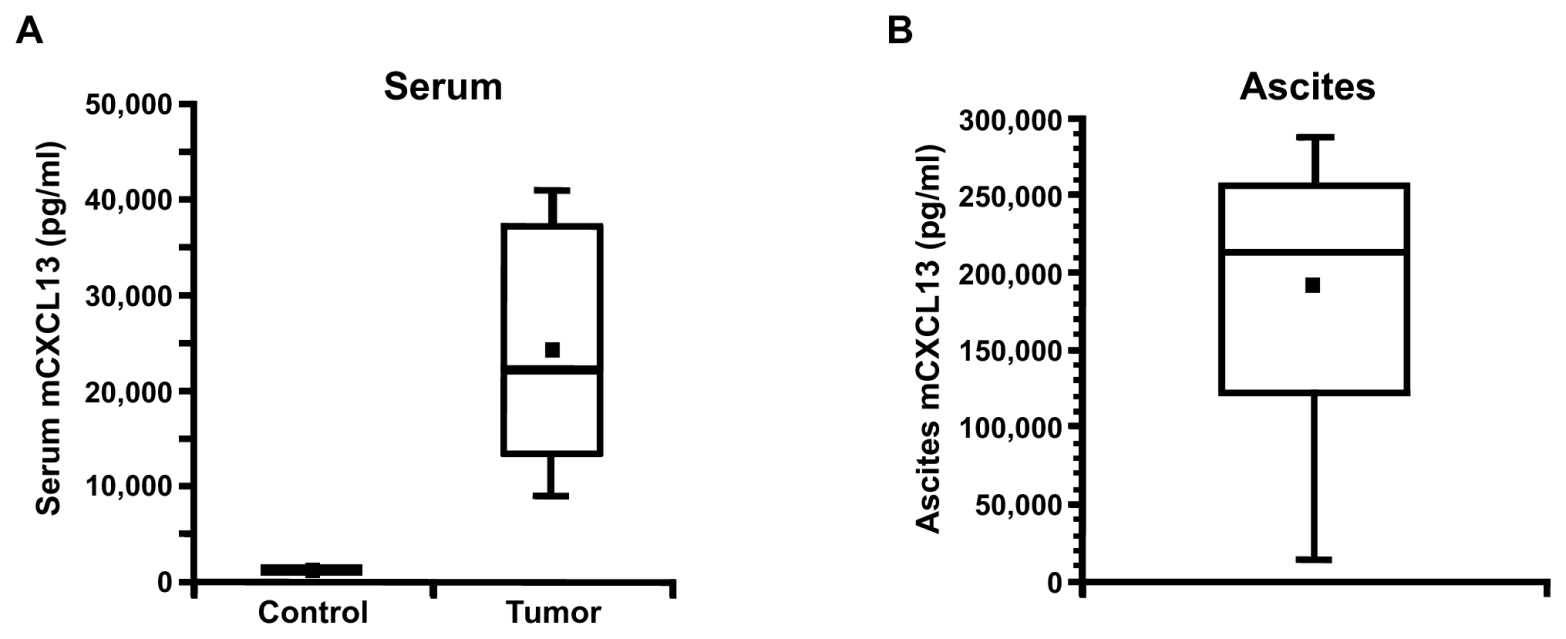
Murine CXCL13 levels are greatly elevated in the serum and ascites of mice with 2F7-derived AIDS-BL tumors. (**A**) Murine CXCL13 levels in the serum of control mice (n = 13) and in the serum of mice with tumors (n = 14), as determined by ELISA. (**B**) Murine CXCL13 levels in the ascites fluid of the mice with tumors. The solid square within each box plot represents the mean value for the data. In (**A**), there is an ~20-fold increase in mean mCXCL13 levels in the serum of mice with tumors compared to control mice (p < 0.0001; t-test). In (**B**), the long lower whisker in the box plot of the data reflects the presence of several low outliers; these lower values were generally seen in animals that developed smaller, more localized tumors at the site of injection as opposed to widespread metastatic disease.

In contrast, serum murine CXCL13 levels were found to have increased ~20-fold in animals that developed tumors (p < 0.00002) (Figure 4A). Murine CXCL13 levels in tumor ascites were generally extremely high, with an average value of nearly 200,000 pg/ml (Figure 4B). These results were replicated in additional independent experiments (not shown). There was a correlation between serum and ascites murine CXCL13 levels in individual mice (Pearson’s correlation coefficient = 0.532, p = 0.049).

Detailed studies of tumors in mice injected with R (AIDS-DLBCL) cells or BCBL-1 (AIDS-PEL) cells were not performed. However, the animals that developed R- or BCBL-1-derived tumors (n = 2 /group) all showed greatly elevated serum levels of murine CXCL13 (ranging from about 10,000 to 40,000 pg/ml), and in tumor ascites fluid (ranging from about 25,000 to 100,000 pg/ml).

As noted in the *Materials and Methods*, an attempt was made to estimate background murine CXCL13 levels in the peritoneal cavity of normal control NOD-SCID mice, which resulted in a low estimate of ~900 pg/ml, and a high estimate of ~9,000 pg/ml. Both of these estimates are much lower than the median murine CXCL13 level seen in the ascites of mice bearing 2F7-derived tumors (shown in Figure 4B).

### Murine CXCL13 is associated with tumor-infiltrating cells of mouse origin in tumors in the 2F7 AIDS-BL model

Expression of human CXCR5, human CXCL13, murine CXCL13, and the mouse histiocyte marker, F4/80, were examined by immunohistochemistry in both primary tumors and metastases from mice inoculated with 2F7 cells as noted in the *Materials and Methods*. Representative results are shown in Figure 2. Tumors typically expressed human CXCR5 (Figure 2B-C), consistent with the flow cytometry results shown in Figure 3. In contrast, a negligible expression of human CXCL13 was generally seen (Figure 2C-D), which is consistent with the results of serum/ascites studies described earlier. Interestingly, both primary tumors (shown) and metastases (not shown) all contained what appeared to be infiltrating, macrophage-like non-tumor cells that strongly stained positive for murine CXCL13 (Figure 2E). To determine what type of cells these were, serial tumor sections were stained using antibodies to the mouse histiocyte marker, F4/80, and for murine CXCL13. As shown in Figure 2F, tumors were consistently infiltrated with F4/80+ cells. Cells that were F4/80+ and cells that were murine CXCL13+ were present in sequential serial sections (Figure 2G-H). Furthermore, both F4/80+ and murine CXCL13+ cells had a phenotype similar to that of a histiocyte, which included the presence of dendritic processes on some cells (Figure 2G-H). These results are consistent with the murine CXCL13+ tumor-infiltrating cells being mouse histiocytes.

### Blocking the interaction of CXCL13 with its receptor, CXCR5, results in a substantial delay in 2F7 AIDS-BL tumor formation in mice

**Figure 5:**
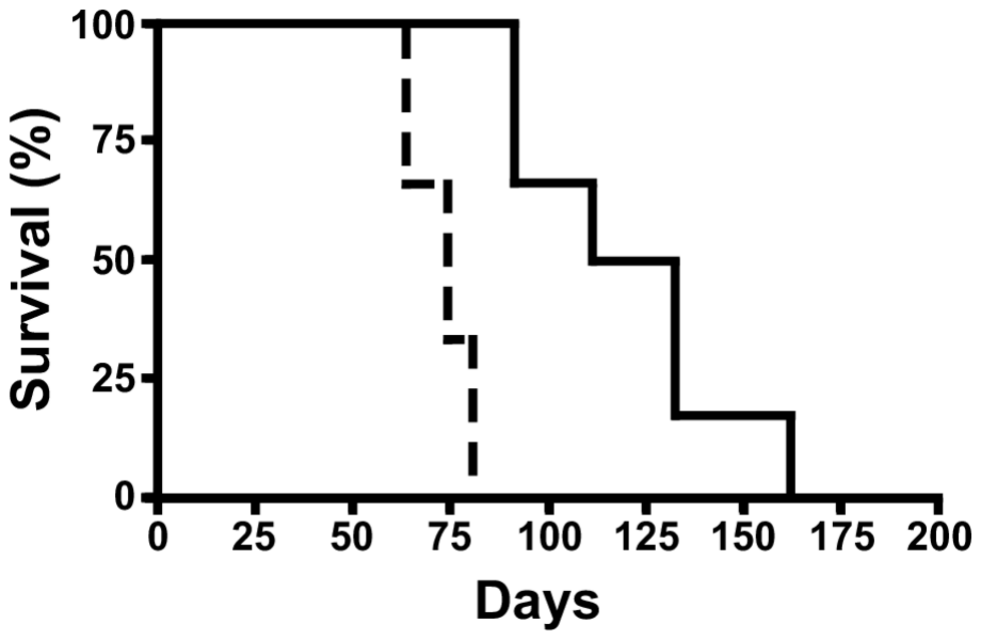
Inhibition of CXCR5 substantially increases length of survival in the 2F7 NOD-SCID mouse model of AIDS-BL. In one group of mice (n = 6), 2F7 cells were pre-incubated with neutralizing antibody to CXCR5 prior to injection into mice. In the control group (n = 6), cells were pre-incubated with an isotype control antibody. The figure depicts a survival curve for the experiment. The number of days elapsing since the injection of 2F7 cells is shown on the x-axis, and the percentage of surviving mice is shown on the y-axis. The solid line depicts results for the anti-CXCR5 test group; the dotted line depicts results for the isotype control group. These results are significant (p < 0.0007, log-rank test). Two additional replicates of this experiment have been performed, with similar results.

To determine if human CXCR5 plays an essential role in the formation of 2F7-derived BL tumors in this model, mice (n = 6) were injected i.p. with 2F7 cells that had been pre-incubated with a neutralizing antibody to human CXCR5, as noted in the *Materials and Methods*. Control animals (n = 6) were injected with 2F7 cells that had been pre-incubated with an isotype control antibody. As shown in Figure 5, tumor formation in the anti-CXCR5 treatment group was substantially delayed, with the median time to death in this group being 120.5 days (95% CI = 90-161 days), compared to 73 days (95% CI = 62-77 days) for the control group (p < 0.0007, log-rank test); this represents a 65% increase in the time to death in the treatment group. Two additional replicates of this experiment were performed, with similar results.

## Discussion

In these studies, a novel mouse/human xenograft model of AIDS-BL was created by injecting NOD-SCID mice i.p. with the AIDS-BL cell line, 2F7. In this model, mice reliably develop tumors in the peritoneal cavity, with metastases to other locations (Figure 1). The development of this model is significant, because to date few tractable animal models of AIDS-related BL exist. Monkeys infected with simian immunodeficiency virus (SIV) will sometimes develop Burkitt-like (BL) lymphomas [24]. However, monkeys are expensive and difficult to work with, and only a small proportion of SIV-infected animals will develop BL-like tumors [24]. Laurence et al. [25] reported that a subset of human B cells could be infected with HIV *in vitro* and would then develop a malignant phenotype, becoming capable of producing Burkitt-like tumors when injected subcutaneously into SCID mice. However, human AIDS-BL tumor cells are typically not infected with HIV [26]. Several mouse/human xenograft models of BL unrelated to HIV infection have been described [27,28]; however, to our knowledge, the expression of CXCL13 been not been reported in these models. In one model of particular interest, NOD-SCID mice formed tumors in the peritoneal cavity that significantly resembled BL after i.p. injection of the Namalwa EBV(+) non-AIDS-associated BL cell line [27]. Interestingly, the chemokine, CXCL12 (SDF-1), and its receptor, CXCR4, played an essential role in development of tumors in this model, as blockade of these molecules abrogated tumor growth [29]. However, expression of CXCR5 or CXCL13 was not examined in that model.

Because both the 2F7 cell line and Namalwa cell lines are similar in that they are EBV(+) BL cell lines, we initially studied CXCR4/CXCL12 in our model, but found no indications that these molecules were important in promoting tumor growth in this model (unpublished results). Instead, surface expression of CXCR5 was seen to be greatly elevated on cells of 2F7-derived tumors in the model (Figure 3). While human CXCL13 was undetectable in serum or ascites fluid from animals with tumors (*Results*, not shown), levels of murine CXCL13 were greatly elevated in these fluids (Figure 4). These findings are significant for the following reasons: first, this is, to the best of our knowledge, the first report of CXCL13 overexpression in any mouse model of BL, HIV-associated or not. Second, these results are consistent with our previously published results that most primary human AIDS-lymphomas, including tumors of the Burkitt subtype, were positive for both CXCR5 and CXCL13 expression, and that serum levels of CXCL13 were elevated in subjects who developed AIDS-lymphoma [18]. The results of the current study thus provide additional evidence that these molecules are overexpressed in AIDS-lymphoma, and that CXCL13 is a potential biomarker for this disease. The overexpression of these molecules in this mouse model also helps validate the model as successfully recapitulating aspects of human AIDS-BL. Third, the fact that high levels of murine, but not human, CXCL13 are present in the serum and ascites fluid of animals with 2F7-derived tumors suggests that non-tumor mouse cells are responsible for CXCL13 production in this model. The immunohistochemistry results (Figure 2) further confirm this, as they indicate that the tumors are infiltrated with mouse cells (probably histiocytes) that show positive staining with antibodies against murine, but not human, CXCL13. Further studies are needed to definitively identify the actual mouse cells producing murine CXCL13 in this model, since the CXCL13(+) tumor-infiltrating cells might merely be binding circulating chemokine, which is present at very high levels. These results raise the possibility that tumor-infiltrating cells could potentially be a significant source of the elevated levels of CXCL13 that are seen in the human disease. Fourth, the fact that differential levels of human and murine CXCL13 are readily seen in the model and are readily identifiable as being produced by tumor (human CXCL13) or non-tumor (murine CXCL13) cells, suggests that this model could have substantial potential utility for determining the source of other molecules that are known to be over-produced in persons who develop AIDS-lymphoma, such as soluble CD23 and IL-6 [30,31]. Similarly, the mouse model should be useful for studies to determine how tumor cells and cells of the immune system interact with each other.

The finding that tumors in the 2F7 model are infiltrated by mouse cells, some of which bear the mouse histiocyte marker, F4/80 (Figure 2F and 2H), and some of which stain positively for murine CXCL13 (Figure 2E and 2G), has additional significance. It has been reported that a substantial proportion of AIDS-lymphomas are infiltrated by histiocyte-type cells, such as macrophages [4,5]. The fact that this is seen in the NOD-SCID mouse model provides additional evidence that the model is able to recapitulate processes occurring in AIDS-lymphoma in humans. This histiocyte infiltration is particularly interesting in view of the work of McGrath et al. [6], who noted that isolated tumor-infiltrating macrophages from AIDS-lymphomas are able to promote tumor formation in SCID mice. The results in the 2F7 NOD-SCID model suggest that the reverse may also be true: AIDS-lymphoma tumor cells may also be able to attract and activate tumor-infiltrating cells, such as histiocytes, which may then possibly potentiate the growth of the tumor cells.

Our results identify CXCL13 as potentially being an important molecule linking the innate immune system and tumor cells in the pathogenesis of AIDS-lymphoma. We have previously reported that 2F7 cells show chemotaxis towards human CXCL13 *in vitro* [18]; others have reported or inferred that murine CXCL13 can act on human CXCR5, and that human CXCL13 can induce proliferation of tumor cells in some cancers [32,33]. The high levels of CXCR5 and murine CXCL13 that are seen in the 2F7 model thus raises the possibility, although does not prove, that murine CXCL13 (possibly produced by tumor-infiltrating cells) acts to promote tumor growth. The fact that pre-incubation of 2F7 cells with neutralizing antibody to CXCR5 substantially delays the time to death in the model (Figure 5) provides additional evidence that the CXCR5/CXCL13 axis could potentially be playing an important role in tumor development in the model. Results from these latter studies should be interpreted with caution, though, since it remains possible that antibody-bound 2F7 cells are being killed in the mice by mechanisms such as antibody-dependent cell-mediated cytotoxicity (ADCC). This possibility seems somewhat unlikely, however, since NOD-SCID mice, in addition to having virtually no B cells and T cells, also have extremely low natural killer cell activity and almost no complement activity, and myeloid lineage cells are defective [34,35]. Clearly, though, further studies will be needed to more precisely determine the role that CXCR5 and CXCL13 may be playing in tumor growth.

Finally, the fact that high levels of murine CXCL13 were also observed in NOD-SCID mice that developed tumors after inoculation of either the R (AIDS-DLBCL) cell line or the BCBL-1 (AIDS-PEL) cell line (*Results*, not shown) suggests that the 2F7 cell line is not atypical of AIDS-lymphoma cell lines, in terms of being able to promote murine CXCL13 overproduction in tumor-bearing NOD-SCID mice. It also suggests that promotion of murine CXCL13 overproduction in NOD-SCID mice is not restricted to AIDS-lymphoma cell lines of the Burkitt subtype. The possibility also exists that overexpression of murine CXCL13 may be a common phenomenon in many NOD-SCID models of cancer, B cell-related or not. Future studies are needed to examine this issue, as it appears that no previous studies on murine CXCL13 expression in NOD-SCID models of cancer appear to have been reported.

In summary, we have established a novel mouse/human xenograft model of AIDS-BL lymphoma using the AIDS-BL cell line, 2F7. In this model, greatly elevated levels of CXCR5 are seen on the surface of tumor cells, and greatly elevated levels of murine, but not human, CXCL13 are seen in the serum and ascites of animals with tumors. These studies suggest that non-tumor murine cells are responsible for the overproduction of CXCL13 observed in this model, and raise the possibility that immune cells and tumor cells may interact via the CXCR5/CXCL13 axis in this model, and potentially, in human AIDS-lymphoma as well. Further studies are needed to more definitively define the role of the CXCR5/CXCL13 axis, both in murine models, and in human AIDS-lymphoma.
